# The effect of perceived organizational support on the prohibitive voice behavior of knowledgeable talents during the COVID-19 pandemic: Exploring moderating role of the digitalization level

**DOI:** 10.3389/fpsyg.2022.1020263

**Published:** 2022-11-23

**Authors:** Xiao Chen, Kangjun Ren, Yan Shen

**Affiliations:** ^1^Talents Office, Zhejiang University of Technology, Hangzhou, China; ^2^School of Management, Zhejiang University of Technology, Hangzhou, China; ^3^Jing Hengyi School of Education, Hangzhou Normal University, Hangzhou, China

**Keywords:** the COVID-19 pandemic, perceived organizational support, knowledgeable talents, prohibitive voice behavior, level of organizational digitization

## Abstract

This study focuses on the effect of perceived organizational support on the prohibitive voice of knowledgeable talents and on the factors influencing that voice during the COVID-19 pandemic in China. Grounding our research in social exchange theory and the Chinese sociocultural background, we collected data from 714 questionnaires from universities in Zhejiang Province and analysize by linear regression and found, first, that perceived organizational support significantly increased the prohibitive voice of knowledgeable talents and, second, that in the relationship between perceived organizational support and the prohibitive voice of those talents, the level of digitalization level of an organization has a positive the effect of perceived organizational support on prohibitive voice behavior. This study enriches the theory of voice behavior, helps universities understand the behavioral patterns of knowledgeable talents and guides the cultivation of an atmosphere with prohibitive voice behavior.

## Introduction

The Coronavirus Disease 2019 (COVID-19) has evolved into a global crisis. In this COVID-19 pandemic age, Chinese people are not only threatened at the physiological health level but also suffered a subtle but more long-lasting impact on the psychological level (Cao et al., 2020; Qiu et al., 2020). The pandemic brought stressors to people, such as reduced income and reduced face-to-face social contact, while stressors are correlated with behavior (Holmes et al., 2020). Meanwhile, the COVID-19 pandemic had a consequence on organizations and their member, and it changed extra-role behavior in the field of human resource management (Manuti et al., 2020) and working modalities practice. Therefore, we are curious about that how will voice behavior, which is one of extra-role behavior, be affected.

Voice behavior is defined as change-oriented behavior aimed at improving the organizational status quo (Van Dyne and LePine, 1998). As one of the proactive behaviors available to an organization member, voice behavior responds to the requirements of new management concepts (such as knowledge management) and has become a focus of theoretical attention. Liang et al. (2012) classify members’ voice behavior into promotive voice and prohibitive voice. Promotive voice behavior expresses members’ ideas and opinions that can improve the organization’s workflow and solve its practical problems; prohibitive voice behavior expresses members’ sense of dissatisfaction with the organization or general protest authority, such as directly pointing out harmful or inefficient behavior, procedures, rules, or policies in the organization. Although both types of voice behavior help organizations improve the quality of decision-making, correct operational deviations, and improve management effectiveness, there are significant differences in the risks and consequences of each.

In fact, compared to promotive voice behavior, which emphasizes constructive ideas and opinions, prohibitive voice behavior, with its defensive nature, is more effective in preventing crises, stimulating trains of thought (Liang et al., 2012), and improving overall organizational safety by challenging the organizational status quo and highlighting threats and risks in the environment in order to strengthen the team’s supervision of loss prevention and drive the team to achieve relevant safety goals (Li et al., 2017), which has a more positive effect on avoiding team crises and is more in line with practical needs (Morrison, 2011). However, under the influence of traditional beliefs such as *the superiority of those at the top and the inferiority of those at the bottom* in China, most management is reluctant to see subordinates challenge existing policies; it is rare for members of the organization to be willing to voice inhibiting ideas because of the sense that *tongue-tied knowledge is preferable to ignorant loquacity*. We, therefore, argue that for organizations to develop better in the Chinese context, they need to be guided out of the dilemma of member reticence. Research on the factors influencing inhibitory talk is particularly important because prohibitive voice behavior is rarely motivated by self-interest (Kim et al., 2013).

In the literature, the effects of various antecedents, including the individual factors (e.g., gender, personality, psychological safety, cultural intelligence) and organizational contextual factors (e.g., management innovation, supervision, organizational climate), on prohibitive voice behavior has been explored in empirical studies (Janssen and Gao, 2015; Guzman and Espejo, 2019; Roch et al., 2019; Engemann and Scott, 2020). However, despite this prohibitive voice behavior related to existing research, we believe current prohibitive voice behavior research needs improvement. First, the early literature has been limited to treating voice behavior as a unidimensional generalization while promotive voice behavior and prohibitive voice behavior have been treated as one. Sometimes the researcher even only focused heavily on promotive voice behavior. Consequently, prohibitive voice behavior remains underexamined, especially its antecedents and underlying psychological mechanisms. Second, only a few existing pieces of research about voice behavior tend to analyze the individual factors and organizational contextual factors jointly (Liang et al., 2012; Engemann and Scott, 2020); only a small number of research focus on individual responses to organizational contextual. Therefore, the reaction from an individual to an organizational context which influences voice behavior needs to go further. Third, from the psychological factor perspective of voice behavior, felt obligation and responsibility for affiliations as an important antecedent of prohibitive voice behaviors has aroused the interest of many scholars.

Perceived organizational support (POS) may be a psychological mechanism of prohibitive voice behaviors based on social exchange theory. Social exchange theory emphasizes that resources in social exchanged through a process of reciprocity, whereby one party tends to repay the good deeds of another party (Chernyak-Hai and Rabenu, 2018). It is now widely used to analyze the relationships between members and the organization (Meira and Hancer, 2021). However, the existing literature focuses more on the interests that two parties can be obtained, such as work performance (Aydın and Kalemci Tüzün, 2019; Kim and Qu, 2020) from an organisational perspective and psychological well-being (Panaccio and Vandenberghe, 2009; Yousaf et al., 2019) from an individual perspective, while overlook importance of the organizational citizenship behavior which makes member contribute spontaneously to the organization. Given prohibitive voice behavior is more likely to come from spontaneous behavior while voice behavior is one of organizational citizenship behavior, we design the research to study how voice behavior, especially prohibitive voice behavior associated with POS based on social exchange theory. When members perceived that they are valued and supported by their organization will raise POS (Eisenberger and Stinglhamber, 2011), and then elicited felt obligation and responsibility who, in turn, reciprocate by helping the organization through the performance of citizenship behavior (Thompson et al., 2020). This process is more likely to be influenced by the degree of perceiving of members rather than the actual support they are received.

Prolonged exposure to the pandemic and strict quarantine measures nationwide encourages all organizations to speed up the process of digitalization in the context of the pandemic and increasing adopt digitalization form in an organization and the office (Amankwah-Amoah et al., 2021), online work has become popular. Generally, people turn to social media platforms and other digitalization platforms (Lewis and Molyneux, 2018) to exchange social support, and express their voice. The digitalization platforms equip people with ‘microphones’ by letting them have a voice in work arrangements and enterprise institution improvements (Gegenhuber et al., 2021). Given the online work has the characteristics of non-face-to-face and people focus on the negative during the Covid-19 pandemic age (Chopdar et al., 2022), the digital level encourages voice behavior at the same POS level, especially prohibitive voice behavior.

For this reason, this paper takes the social exchange theory as the theoretical framework and tries to find out the relationship between perceived organizational support and the prohibitive voice. This paper may have the following contributions: on the one hand, this paper advances the research on perceived organizational support with a focus on members’ voice behavior, which enhances understanding of the behavioral motivation for members’ voice behavior. Previous studies overgeneralize voice behavior and overlook the mechanism of action of prohibitive voice behavior. In this paper, we focus on prohibitive voice behavior and introduce social exchange theory into research. It enriches the literature of social exchange theory and broadens the application scenarios in the social exchange theory field. On the other hand, this paper takes the response of organizational members to digitization into consideration. It enriches the literature about the impact of digitization during the COVID-19 pandemic while the research about a member’s response to an organization’s digitalization is booming. Besides, this paper also provides a strategy for guiding universities out of the “silence is golden” voice atmosphere during the pandemic age.

## Literature review

### Perceived organizational support

In the early days, when talking about the relationship between organizations and members, large amounts of research emphasized members’ attitudes and commitment to the organization rather than the organization’s support to its members. It was not until 1986 that Eisenberger and Huntington combined social exchange theory, reciprocity principles, and organizational anthropomorphism to propose perceived organizational support (POS), going on to fill in the related research gaps. When members feel supported and affirmed by an organization, they give the organization more positive feedback in return. Employee engagement, job satisfaction, and organizational commitment.

The scholars studied antecedents and outcomes of perceived organizational support. Ahmed and Nawaz (2015) proposed that an organization where members feel supported makes them reciprocate it spontaneously by offering attitudinal and behavioral outcomes while the supporting of an organization is antecedent and behavior of members is outcomes. Aydın and Kalemci Tüzün (2019) proposed that there is a strong relationship between POS with work performance. Kim and Qu (2020) came up with similar conclusions to Aydın and Kalemci Tüzün (2019) in their research. Fukui et al. (2019) concluded that POS will decrease turnover intentions. Chen and Eyoun (2021) considered COVID-19 and researched job insecurity, emotional exhaustion, and POS. These authors concluded that POS can help employees overcome their insecurities and moderate emotional exhaustion. Xu and Yang (2021) surveyed teachers in primary and secondary schools and demonstrated that POS influenced teaching exhaustion and inefficacy. However, the few existing empirical research on POS widen the research field to professors at colleges/universities (Oubibi et al., 2022).

### Voice behavior

Within the existing studies, voice behavior is now typically categorized as task-based participation (Wilkinson et al., 2015 ), upward problem-solving (Jin and Wu, 2021), formal grievance procedures (Mowbray et al., 2014), downward communication (Hylving and Bygstad, 2019). Kim et al. (2013) show that all three motives, including preserving one’s image and interests, prosocial motives, and reserving organizational interests, contribute to organizational citizenship behavior. Lemmon and Wayne (2015) proposed egoistic and altruistic motivations for organizational citizenship behavior. Voice behavior is considered an act of citizenship to the organization (Chiaburu et al., 2013). It’s an extra-role behavior in which individuals express their views or concerns about work-related issues (Morrison, 2011; Mowbray et al., 2019), and it is not coercive. In general, those motivations mentioned above may be relevant to voice behavior.

Liang et al. (2012) categorized voice behavior into promotive voice behavior and prohibitive voice behavior. Promotive voice behavior indicates improvements in work practices and procedures, focuses on future matters, is primarily concerned with achieving the vision of the constructors, and is designed to make improvements to existing work practices and management innovation (Guzman and Espejo, 2019); prohibitive voice behavior focuses on the expression of warnings about factors that can harm the organization and tends to identify concerns about existing or potential harm to the organization (Liang et al., 2012; Kakkar et al., 2016). Prohibitive voice behavior is seen as more of an “oppositional behavior” for members, as most leaders do not want to see the policies and rules that they have set questioned or challenged by their subordinates (Chamberlin et al., 2017). Morrison (2011) suggested that members are more likely to choose silence for fear of unfair treatment by the power hierarchy; only when they feel that they are valued by their supervisors and will not be punished for voicing concerns is information likely to *flow from the bottom to top*. Kakkar et al. (2016) proposed that the avoidance orientation of members have a positive relationship with prohibitive voice behavior.

### Knowledgeable talents

Kianto et al. (2019) argue that “knowledgeable talents” possess intelligence, rationality, and responsibility, which involve effort and improvisation in creating and applying knowledge. Chinese scholar Zhang Qianqiang summarizes many special characteristics of knowledge-based talents and suggests that knowledge-based talents are distinguished by being of higher professional quality innovative, more mobile, and capable of greater labor complexity (Zhang, 2009). Focusing on the organizational scenario of Chinese universities, this paper argues that university teachers meet these four characteristics to a certain extent. First, university teachers are generally of high professional quality. They have received systematic professional education, have a higher education level, a higher knowledge level, relatively open minds and vision, good learning, and comprehension ability, etc. Second, university teachers are innovative. They use their rich theoretical knowledge to engage in creative work and constantly add to the body of knowledge. Third, university teachers have high mobility. They possess special production factors, i.e., knowledge, scientific research results, invention patents, etc., and could accept the challenges of new work and new tasks, giving them far more career options than traditional workers. Fourth, the labor of university teachers has high complexity. They are mainly engaged in thinking activities, and the results of their labor are mainly innovation and scientific research results, usually in the form of ideas, creativity, technical inventions, etc. In summary, university teachers meet most of the criteria for being knowledge-based talents and in fact are typical knowledge-based talents. Therefore, the sample of university teachers used in this paper is a representative and reasonable choice for university teachers research on the prohibitive voice of knowledgeable talents.

### Digitization level of the organization

Panic over the pandemic and unflagging strict quarantine measures nationwide get people used to work online and urge all organizations to further embrace digitalization (Amankwah-Amoah et al., 2021; Chopdar et al., 2022).

Digitization can be understood as the conversion from analog information to digital (computer-readable) information (Flyverbom, 2019) or the direct input of digital information (Bailey et al., 2012). As times have changed, organizations in all industries have begun to move toward digital forms of work (Li et al., 2022). Leonardi and Treem (2020) proposed that digitization transforms organizational operational processes by removing or reducing work-related constraints, including those of time, space, and location. As soon as organizations begin to take advantage of the digital nature of work, they have already begun the process of digitization, including new forms of work organization (Hinds and Kiesler, 2002; Wu, 2018; Jin and Wu, 2021), the use of Office Automation (OA) systems, and the operation of knowledge management systems (Alavi and Leidner, 2001). The process of digitization is necessary for contemporary organizations to maximize innovation and remain competitive (Hinings et al., 2018).

Digitization has a broad and far-reaching impact on organizations, and existing research has begun to focus on the reactions of organizational members to digitization. Kuusisto (2015) states that digitization can lead to faster and more comprehensive transfer of information at the disposal of organizational members, including those at lower levels. Vuori et al. (2019) argue that digitization can increase productivity and efficiency. Cijan et al. (2019) show that digitization can increase organization members’ job satisfaction and promote greater member autonomy.

In addition, different digitization level can cause different effects. Kensbock and Stöckmann (2021) focus on members’ perceptions of digital transformation and conduct an analysis of voice behavior, suggesting that digitization motivates organizational members’ voice behavior but that members are less likely to engage in voice behavior once they feel they are being monitored due to participating in the digitization process. Using a sample from the hospitality industry, Romero-Martínez and García-Muiña (2021) concludes that high levels of digitization are beneficial to anti-corruption behavior. In a study on the public sector, Davies and Fumega (2014) find that the higher a sector’s level of digitization, the more responsibly and efficiently members in that sector tend to work.

## Theoretical analysis and hypothesis

### Relationship between POS and the voice behavior of knowledgeable talents during the pandemic age

Manuti et al. (2020) verified the COVID-19 pandemic will influence extra-role behavior and the study of human resource management during the epidemic situation has been taken seriously. Reporting on their 2013 study, Kim et al. point out that members promote extra-role behavior out of the motive of maintaining organizational interests, a case of prohibitive voice. Based on the general principles of social exchange theory, when knowledgeable talents are valued by an organization’s management or find their values reflected in the organization will improve their level of POS. The logic of POS is derived from social exchange theory which explains human behavior and the social structure of its relation (Kim and Qu, 2020).

The social exchange theory is now widely used to analyze the causal relationships between members and the organization (Meira and Hancer, 2021). According to this theory, members and organizations can be seen as two parties in an exchange, and where individuals tend to reciprocate with positive behavior to an organization when they receive positive treatment or feel valued by others (Chernyak-Hai and Rabenu, 2018). It is well documented that POS increases members’ commitment to the organization (Kim et al., 2016), and motivates members’ engagement (Aydın and Kalemci Tüzün, 2019). At the same time, POS has a positive effect on members’ well-being (Caesens et al., 2017) and other extra-role behaviors (Alnaimi and Rjoub, 2021). Wang and Hsieh (2013) argued that managers should nurture a supportive organizational ethical climate for members to raise POS and then ameliorate employee silence. Likewise, Men and Yue (2019) suggest that POS has a significant positive effect on members’ willingness to communicate. Thus, POS may increase prohibitive voice behavior.

On the one hand, voice behavior as one of organizational citizenship behavior will be affected by POS based on previously discussion. Prohibitive voice behavior is more likely come from spontaneous behavior which is one of the organizational citizenship behaviors. Therefore, prohibitive voice behavior is likely to relevant to POS. The COVID-19 pandemic has caused some consequences in knowledgeable talents’ psychology that people become more negative and pessimistic (Lades et al., 2020). These encourages knowledgeable talents dare to provide a prohibitive voice. At the same time, knowledgeable talents usually have strong independence. They are extremely eager to be respected and recognized by the organization and even the society, and they have a strong desire to perform, which makes them more likely to make use of prohibitive voice behavior.

On the other hand, it leads knowledgeable talents to develop a strong sense of belonging and “ownership” and a sense of obligation to repay and thank the organization for the POS. Especially, members may be in a great panic during the COVID-19 pandemic. It is critical to make members feel cared and relieve members’ pressure as well as increase their confidence in being well treated, well valued and well recognized (Cao et al., 2020; Qiu et al., 2020). Knowledgeable talents in this situation do their best to complete all work-related tasks, give advice to the organization’s management, and put forward their considerations about existing or possible problems facing the organization, which is called “*picking holes*.” They do so in the hopes that not only will top management perceive their loyalty, pay attention to the problems they have found, and prepare for the inevitable but the enterprises which fought against COVID-19 with them could pull through. Therefore:

*Hypothesis 1:* POS has a significant positive effect on the prohibitive voice behavior of knowledgeable talents.

### Moderating effect of the digitization level of the organization

COVID-19 pandemic encourages all organizations to speed up the process of digitalization in the context of pandemic (Amankwah-Amoah et al., 2021), the level of digitalization has improved significantly (Raimo et al., 2021).

Digitization improves the flow of information in an organization, gives individual members access to more cutting-edge information, promotes information sharing and creation, and gives members access to more resources (Kensbock and Stöckmann, 2021). Digitization makes members feel cared for and better integrated into the organization, increases members’ POS (Zhao et al., 2021) and might promotes prohibition by members according to the logic of our research. From a sociocultural perspective, “*relationships, face, and favors*” and “*collectivism*” are Chinese cultural concepts that have an important influence on Chinese people’s thinking and behavior (Wu, 2018; Wu et al., 2021). Chinese people prefer to get along with each other smoothly and cordially; they do not like to dismiss others in public and therefore always express their opinions euphemistically (Wu et al., 2022). The traditional form of voice behavior can be divided into two ways: real-name voice behavior or anonymous voice behavior. As we mentioned above, adopting a real-name voice behavior can attract attention and voice tellers will certainly take this into account; while anonymous messages may make voice tellers worry about exposing themselves to others because of handwriting or the process of speaking anonymously being seen by others. Therefore, even when members feel sufficiently supported by the organization, although they may be motivated to give back to the organization, it is difficult for them to be completely “*straightforward*.” The application of digitization has changed the way organizations collaborate and communicate with each other (Dougherty and Dunne, 2012), allowing members to voice concerns through digitization platforms without attracting attention rather than engaging in the traditional form of voice behavior. Gegenhuber et al. (2021) compared the digitization platforms to microphones and suggest that the organization’s digitization encourages employee voice. The more digital the organization and the more confidential the information, the more functional the channel and the less the knowledgeable talents are concerned about repercussions from making suggestions.

Given the online work has the characteristics of non-face-to-face and people focus on the negative during the Covid-19 pandemic age (Chopdar et al., 2022), digital level encourages voice behavior at the same POS level, especially prohibitive voice behavior. There is a series of digital technologies used to effectively combat the unprecedented COVID-19 pandemic in China, especially in educational field of China (Oubibi et al., 2022). Although different universities have different levels of sensitivity to digitalization, it is much more digital than it used to be. This phenomenon provides us a good chance to study moderating effect of digitalization level.

Working in university, knowledgeable talents who feel the same level of organizational support but enjoy a more highly digitized work setting are more able to engage in otherwise prohibitive voice behavior. Therefore, we hypothesize that:

*Hypothesis 2:* The higher the digitization level of the organization, the stronger the positive effect of POS on the prohibitive voice behavior of knowledgeable talents.

## Main study

### Sample and data

This paper focuses on the effect of POS on the prohibitive voice of knowledgeable talents and a mechanism influencing that effect. Survey data were collected from universities in Zhejiang, China. The questionnaire survey design involved two stages, each at a different point in time. A pre-study (time 1) took place from April to May 2021. Paper-and-pencil surveys were distributed to 44 teachers from universities in Zhejiang Province, and 36 were collected on the spot. After removal of four incomplete questionnaires, 32 valid questionnaires remained, for an effective recovery rate of 72.73 percent. During this period, we asked the respondents’ specific opinions on the questionnaire design and their feelings about filling out the questionnaire. Then, confirmatory factor analysis, reliability, and validity analysis were conducted on the 32 collected valid questionnaires. The second phase (time 2) constituted the formal research, conducted between June and August 2021. The research team used Sojump, a professional questionnaire platform, to distribute 900 questionnaires to teachers at universities in Zhejiang. Of these, 721 questionnaires were returned. Excluding incomplete and logically contradictory questionnaires, a total of 714 valid questionnaires were obtained, resulting in a valid recovery rate of 79.33 percent.

The final sample pool yielded 440 male and 274 female participants. As to age structure, 53.36 percent of the respondents were between 31 and 40 years old, and 30.67 percent were between 41 and 50 years old, indicating that there were more young than middle-aged intellectual talents in the sample pool. In terms of degree, 80.95 percent of the respondents had obtained a doctorate, and 18.21 percent of the respondents had a master’s degree. Regarding titles, 47.9 percent of the respondents had intermediate titles, 29.69 percent had deputy senior titles, and only 3.5 percent had junior titles or no titles. In terms of positions, 81.37 percent of the respondents were in teaching and research posts. As for sources of talents, Ordinary talents who graduated from domestic universities accounted for 70.17 percent of the sample, talents who graduated from domestic universities with provincial title comprised 12.32 percent, talents who graduated from domestic universities with national title accounted for 3.22 percent and overseas talents accounted for 14.29 percent.

### Models and measures

To verify whether there is a linear relationship between POS and the prohibitive voice of knowledgeable talents, a multiple regression test was conducted on the relevant data of the questionnaire (Xu and Wu, 2022; Jin et al., 2022) with the prohibitive voice of knowledgeable talents as the explained variable and the POS as the explanatory variable. The multiple regression model was as follows:


(1) $$\begin{array}{l} \mathrm{Voice}=\alpha_{0}+\alpha_{1}POS+\alpha_{2}\mathrm{Gender}+\alpha_{3}\mathrm{Age}+\alpha_{4}\mathrm{Title}\\ +\alpha_{5}\mathrm{Experience}+\alpha_{6}\mathrm{Tenure}+\alpha_{7}\mathrm{Degree}+\varepsilon \end{array}$$

In this model, voice is measured by text length (the total number of words used to express a prohibitive voice). Since POS and digitization level are measured on a five-point Likert scale which was recommended by Gupta (2019) and Leonardi and Treem (2020), and we average the responses, respectively, according to the previous research paradigm; the higher the title, the higher the value; gender is represented by the value of 1 for male and 0 for female; age is represented by assigning 1, 2, 3, 4, 5, or 6 to each age group; experience is the length of time in the workforce, tenure is the length of time in the university, the degree is the highest degree, and the longer the time and the higher the degree, the higher the value. Table 1 presents a complete list of items.

**Table 1:** Measurement items for the study variables.

**Measurement items**
**Explained variable**
*Voice*
I think there are the shortcomings as follows in my work unit.
**Explanatory variable**
*POS*
In my work unit, I can play a personal role well.
During COVID-19, the talent departments of my work unit offer many personal policies.
In my work unit, management values and treats talent work positively.
**Moderating variable**
*Digital Level*
The digital level of organization in my work unit is high.
**Control variables**
*Title*
My title is as follows [options]
*Gender*
My gender is as follows [options]
*Age*
My age is as follows [options]
*Experience*
The year I joined the workforce is as follows [options]
*Tenure*
The year I joined my present work unit is as follows [options]
*Degree*
My highest degree is as follows [options]

## Results

### Confirmatory factor analyses

Before testing our hypotheses, we evaluated the distinctiveness of the study variables through a series of analysis procedures using SPSS. The explanatory variable, POS for knowledgeable talents, was measured by five-point Likert scale questions with a Cronbach’s alpha value of 0.908, which is greater than 0.7, indicating that the variable has high reliability. Exploratory factor analysis (Eigen value greater than 1) was conducted on the three POS items with KMO values of 0.702, which is greater than 0.6 and passed Bartlett’s sphericity test, implying that the variables have good validity. The cumulative variance explained (after rotation) was 75.128 percent, and the factor loadings are shown in Table 2; they have unidimensional characteristics, and each of them is greater than 0.5. The correspondence between the factors and the question items is consistent with the expected results, indicating good validity. To avoid confounding results due to incorrect models, this paper conducted confirmatory factor analyses (CFA) on the variable POS using SPSS software to confirm construct validity. The AVE value was 0.633, which was greater than 0.5, and the CR value was 0.838, which was higher than 0.7, implying that the data in this analysis had good convergent validity.

**Table 2:** Factor loadings for confirmatory factor analysis of POS.

**Item**	**Loading**
In my work unit, I can play a personal role well.	0.902
During COVID-19, the talent departments of my work unit offer many policies.	0.858
In my work unit, management values and treats talent work positively.	0.839

### Descriptive statistics

Table 3 presents the descriptive statistics for the variables in our study. The results of the descriptive statistics show that the mean number of words used in prohibitive voice expressions by knowledgeable talents was 16.64 with a standard deviation of 21.99, which indicates that there were vast differences in prohibitive voice behavior among the talents; the mean value of POS is 2.57, which indicates that most talents perceived only an average level of perceived organizational support. The results of correlation analysis show that the correlation coefficient between prohibitive voice behavior and perceived organizational support of knowledgeable talents was 0.157 and highly significant at the 1% level, indicating a positive correlation between the two (the higher the organizational support perception of knowledgeable talents, the more words of prohibitive voice behavior were published), which initially verifies the validity of hypothesis 1.

**Table 3:** Means, standard deviations, and correlations among variables in the main study.^a^

Variable	Mean	S.D.	1	2	3	4	5	6	7	8
1. Voice	16.640	21.990	1							
2. POS	2.570	0.770	0.157***	1						
3. Title	2.620	0.870	0.053	−0.054	1					
4. Gender	0.620	0.490	0.096**	−0.054	0.197***	1				
5. Age	2.370	0.760	−0.035	0.099***	0.512***	0.017	1			
6. Experience	3.250	1.340	−0.072*	0.120***	0.534***	−0.043	0.826***	1		
7. Tenure	2.930	1.240	−0.086**	0.163***	0.416***	−0.065*	0.730***	0.872***	1	
8. Degree	2.800	0.420	0.100***	−0.082**	0.082**	0.189***	−0.375***	−0.471***	−0.480***	1

^a^*N* = 714.

*p < 0.1, **p < 0.05, ***p < 0.01.

### Multiple regression analysis: Basic regression results

Firstly, the VIFs (1.05, 1.89, 1.09, 3.24, 6.99, 4.36, 1.65) and the mean VIF (2.90) of the respective variables were calculated. These were less than 10, indicating that there was no multicollinearity problem among the variables. Secondly, the White test results showed that the value of p was equal to 0.000, so there was a heteroskedasticity problem. In doing so, the t statistic may be inaccuracy and further influence the result. According to the research studied by Chiaburu et al. (2015) and Zhang et al. (2021), we choose the WSL to address the heteroskedasticity problem and the regression results are shown in Table 4.

**Table 4:** Basic regression results.

**Variable**	**Prohibitive voice behavior**		
	**OLS**	**OLS**	**WLS**
POS	4.472***	5.489***	5.058***
	(4.25)	(5.16)	(4.48)
Title		3.060**	3.650***
		(2.41)	(2.61)
Gender		3.093*	3.173*
		(1.80)	(1.68)
Age		1.175	1.236
		(0.61)	(0.59)
Experience		−1.481	−3.031*
		(−0.94)	(−1.75)
Tenure		−1.898	−1.380
		(−1.40)	(−0.90)
Degree		0.748	0.589
		(0.30)	(0.22)
Constant	5.139*	−1.900	1.540
	(1.82)	(−0.23)	(0.17)
Observations	714	714	714
*R*-squared	0.023	0.050	0.050

*t*-statistics in parentheses.

*p < 0.1, **p < 0.05, ***p < 0.01.

The OLS results without adding control variables showed that the regression coefficient between voice and POS was 4.472 and highly significant at the 1% level. The results after adding all control variables to the OLS model showed a significant positive correlation between voice and POS at the 1% level. The result shows that POS has a positive effect on prohibitive voice behavior. It is in line with Wang et al. (2020), Meira and Hancer (2021), and Bergeron and Thompson (2020). Meanwhile, the WLS results showed a significant positive correlation between voice and POS at the 1% level. The above results show that POS significantly increases the prohibitive voice behavior of knowledgeable talents, and hypothesis 1 is verified. This indicates that in the context of Chinese society when there is high POS, knowledgeable talents in universities actively contribute to the organization and “*picking holes*.”

### Heterogeneity analysis

The relationship between POS and the prohibitive voice behavior of knowledgeable talents may be influenced by other factors. Research on digitization has become more and more popular, especially during the COVID-19 pandemic (Barrett et al., 2015; Solís García et al., 2021). Therefore, we wonder whether the digitization level of the organization plays a role in prohibitive voice behavior. We use the item “The digitization level of organization in my work unit is high” to group the samples into regressions, and those who choose “very high” and “relatively high” are regarded as indicating a high digitization level of the organization (Digital = 1), while the rest are regarded as indicating a low digitization level of the organization (Digital = 0). We used the seemingly unrelated regress estimation (SURE) and Chow test and the results are shown in Table 5.

**Table 5:** Results of heterogeneity analysis.

**Variable**	**Voice**	
	**Digitization = 0**	**Digitization = 1**
POS	1.342***	13.311***
	(−0.097)	(−3.320)
Controls variable	YES	YES
Constant	2.396	7.410
	(−0.830)	(−0.220)
Observations	577	137
*R*-squared	0.04	0.111
SURE	0.041**	
Chow test	0.000***	

*t*-statistics in parentheses.

*p < 0.1, **p < 0.05, ***p < 0.01.

The results calculated by SURE are 0.041 and highly significant at the 5% level while it is highly significant at the 1% level based on the Chow test. The results mean that the influences of POS are significantly different in the high and low digitalization groups. The coefficient of POS in the high digitalization group is higher than in the low digitalization group. It means POS have a more positive effect on prohibitive voice behavior. The effect of POS on prohibitive voice behavior is stronger if the organization has a higher digitalization level of organization. The perception of digitalization will subtly influence human behavior. This conclusion is in line with the research study by Kim and So (2022). Given the context of Chinese society, on the one hand, since POS may make knowledgeable talents feel obligated to repay the organization (Oubibi et al., 2022), that may motivate them to engage actively in voice behavior. Yet on the other hand, as they are influenced by traditional concepts such as “*superiority and inferiority*” and “*human feelings and face*,” it is very difficult for knowledgeable talents to voice concerns. However, the digitization of the organization gives knowledgeable talents a digitalization channel that favors voice behavior.

The traditional route of voice behavior from the lower level to the higher level requires many intermediate links and steps, and these links and steps can cause members to worry about whether their prohibitive voice behavior will damage their careers (Kakkar et al., 2016). Given such misgivings, members are likely to provide a prohibitive voice when feeling responsibilities and obligations (Thompson et al., 2020) and contribute spontaneously to the development of the organization or they are willing to work with the organization through thick and thin.

When organizations are digitized, knowledgeable talents are less worried about voice behavior, which is also easier and faster for them. So, they are more motivated to engage in prohibitive voice behavior. In addition, the higher the level of digitization, the more channels there are for anonymous voice behavior. Therefore, knowledgeable talents who feel the same level of POS and who work in organizations with higher levels of digitization show a higher sense of obligation to reciprocate and are thus more motivated to engage in prohibitive voice behavior.

## Conclusion and inspirations

This paper empirically examines the relationship between POS and the prohibitive voice behavior of knowledgeable talents using 714 questionnaires administered to university teachers in the pandemic age in Zhejiang, China. COVID-19 pandemic makes China an appropriate social laboratory for examining the correlations between POS and prohibitive voice behavior and the digital level of the organization in the context of pandemic. The results show that (1) POS significantly increases the prohibitive voice behavior of knowledgeable talents (2) the digital level of the organization affects the relationship between POS and the prohibitive voice behavior of knowledgeable talents. Specifically, the positive effect of POS on prohibitive voice behavior is enhanced if the digital level of the organization is high.

Based on the research in this paper, we offer the following points of inspiration. First, in the knowledge economy, members are valued not only for their working ability but also for their ability to generate creative ideas and perspectives. As the complexity of the organizational environment and the intensity of competition increase, listening to and valuing members’ voices becomes critical to the survival and growth of the organization. Especially during the pandemic age, people are anxious and impetuous (Lades et al., 2020) and tend to express their concerns. Therefore, modern organizations need to create a welcoming atmosphere for members’ voice behavior within the organization. Management needs to actively listen to members’ voices and pay special attention to what they communicate through the prohibitive voice. A prohibitive voice also reveals the hidden problems of the organization. If management does not pay attention, such problems are likely to develop into devastating factors for the organization. A prohibitive voice also reveals factors that cause members dissatisfaction. Such factors are likely to stimulate members’ negative emotions, which is detrimental to the organization’s sustainable development if a long time passes without a response to the members. Therefore, management should judge the suggestions objectively and fairly, rewarding members who make appropriate suggestions rather than considering inhibiting suggestions as “deviant behavior” by members (Wu et al., 2021; Wu, 2018).

Second, POS stimulates a strong sense of appreciation, indebtedness, and gratitude. It makes members feel the presence of the enterprises they work for because of fighting against COVID-19 with them. Members may also use prohibitive voices to express their loyalty or appreciation by “*picking holes*.” This is in line with Herzberg’s “two-factor theory” (Biswas and Bhatnagar, 2013) which states that organizations should give members as much organizational support as possible to meet the needs of the hygiene factor and eliminate members’ dissatisfaction. After that, organizations should find ways to improve motivation factors, which can greatly inspire members to work and cause more information to flow from bottom to top.

Third, management should strengthen the organization’s digital work support, improve the digitalization level of the organization, and make full use of digital advantages to improve the atmosphere surrounding voice behavior. China has cultural roots that emphasize “*middle-of-the-road*” and “*collectivism*,” and members tend to maintain close interpersonal relationships with colleagues and supervisors. Members will only do their best to share a prohibitive voice if they feel sufficiently supported by the organization and are willing to work with it. COVID-19 gradually has a certain impact on knowledgeable talents’ psychology and makes knowledgeable talents tend to voice using digital platform, such as media and OA. Besides, digitization allows knowledgeable talents to voice concerns anonymously while maintaining close interpersonal relationships with others, satisfying their inner sense of obligation to repay the organization without fear of damaging their own interests. It is worth mentioning that management needs to respond to prohibitive voices and provide incentives for reasonable prohibitive voices, to create an atmosphere in which members can contribute prohibitive voices and management can face them.

## Limitations and future research

This paper also has some limitations. First, we acknowledge the limitation in terms of the measurement of the variables. In the present paper, the digitalization level is only split into high level and low level and the items only briefly analyze the level of members’ perception of digitalization. In the future, we may introduce a comprehensive index based on a newly designed questionnaire to fit the research situation. Second, the geographical limitation of data collection would affect the generalizability of the results. Future research could collect large-scale samples from more provinces so that the sample can be more representative of the Chinese context. Third, we fail to compare the research content between pre-pandemic and post-pandemic age because of cross-sectional data. Future research can draw another study (longitudinal study) for temporal comparison by a long-term survey.

## Data availability statement

The raw data supporting the conclusions of this article will be made available by the authors, without undue reservation.

## Ethics statement

Ethical review and approval were not required for the study on human participants in accordance with the local legislation and institutional requirements. The patients/participants provided their written informed consent to participate in this study.

## Author contributions

All authors listed have made a substantial, direct, and intellectual contribution to the work and approved it for publication.

## Conflict of interest

The authors declare that the research was conducted in the absence of any commercial or financial relationships that could be construed as a potential conflict of interest.

## Publisher’s note

All claims expressed in this article are solely those of the authors and do not necessarily represent those of their affiliated organizations, or those of the publisher, the editors and the reviewers. Any product that may be evaluated in this article, or claim that may be made by its manufacturer, is not guaranteed or endorsed by the publisher.
